# Searching for a sustainable solution to increasing chemical pollution

**DOI:** 10.1007/s11356-024-33857-y

**Published:** 2024-06-07

**Authors:** Joanna Rakowska

**Affiliations:** Institute of Safety Engineering, Fire University, Warsaw, Poland

**Keywords:** Chemical pollution, Effects of climate change, Technological disasters, Natural disasters

## Abstract

The growing world population and the development of civilization put pressure on the environment. Irreversible climate changes, biodiversity loss, ocean acidification, land and water degradation, and food scarcity took place. Climate changes affect human health through extreme atmospheric phenomena or indirect effects of ecosystem disruption. The intensification of natural disasters increases the risk of technical failures, and the growing production and release of larger quantities and more new chemical compounds, with different hazard potentials, exceeds the environment’s adaptability and societies’ ability to monitor changes and conduct safety assessments. The article reviews the knowledge and approach to the possibility of reducing the risks and effects of events resulting from chemical pollution. As stated, prevention of further environmental degradation and increased preparedness for natural disasters caused by climate change is critical to public safety and requires contingency plans to continuously adapt to the changing frequency, intensity, and scale of natural disasters.

## Introduction

Human civilization is exposed to various natural hazards that can cause disasters classified according to the type of trigger (Provitolo 2009; Caldera and Wirasinghe 2022). A disaster is a major disruption to a community that causes widespread human, economic, or environmental loss and impacts that exceed the ability of the affected community to recover from its resources (Somasundaram et al. 2007; Margus et al. 2023).

Until the 1970s, disasters were seen as effects caused by natural phenomena. The tragic consequences of failures that occurred in industrial plants around the world in the second half of the twentieth century drew attention to technical and technological hazards as factors triggering industrial disasters. In addition, it also takes into account the fact that social behaviour can also be the cause of extreme disturbances in the functioning of societies. Therefore, the current approach considers natural, industrial, or social behavioural disasters. In addition to these main categories, intermediate elements are distinguished; e.g., Natech is a technological accident caused by a natural hazard involving the release or potential release of hazardous substances (Suarez-Paba et al. 2019; Krausmann et al. 2019). Numerous studies conducted over the last few decades have shown that there is an increase in the frequency and severity of Natech events, which are considered to be the result of the development of societies operating in an environment that is a critical element of the process (Monte et al. 2021).

One of the problems that can occur during both natural and anthropogenic disasters is the presence (release or formation) of hazardous chemicals. These events may cause threats not only to the environment but above all to the life and health of society. There is increasing evidence of cognitive, reproductive, and developmental disorders and premature deaths caused by chemical contamination of the human environment (IARC 2012; Baan et al. 2019; Patel et al. 2020; Naidu et al. 2021; Fuller et al. 2022) and their consideration is extremely important in the process of ensuring public safety. Chemical pollution is an anthropogenic interference with the climate system which can be considered at the level of regional threats or for specific environmental systems (Zhang et al. 2018).

Due to the potential impact on society, the economy, and the environment, disasters are the subject of scientific research in many fields. Hence, disaster risk reduction methods should be developed interdisciplinary. An example is the approach to research on global climate change and its impacts that requires cross-discipline communication as its content includes both social and physical or biochemical aspects (Suarez-Paba et al. 2019), (Monte et al. 2021).

Mechanisms for preventing and responding to crises and disasters must therefore flexibly take into account the challenges of the twenty-first century in terms of security and civil protection.

## Methodlogy

The literature used in this review was taken from several scientific databases, including Scopus, Web of Science, PubMed, and websites. The search string is (“chemical pollution*” OR “chemicals” OR “planetary boundary”) AND (“environment*” OR “disaster” OR “climate change”) in the title, abstract, or keywords of documents.

A search was conducted in English-language publications. These included scientific articles, review articles, book chapters and case reports, reports published after 2013, and legal documents. An initial search of these databases on February 10, 2023, yielded 5045 articles, chapters, and other documents. However, a quick search of the titles of the articles found showed that a large percentage of the articles were related to medical research or considered one type of contaminant. Therefore, we used the database filtering feature to exclude these articles and 3558 documents are omitted. The next step was to reject publications regarding monitoring methods of pollutants. In the end, 118 works remained in the database and 11 articles were removed from the study due to duplication. The author reviewed the abstracts of the documents to ensure their suitability. After this selection, another 28 articles were removed. Based on the references of the analyzed publications, eight more articles have been added.

After this process, 86 English-language articles and documents published in the world’s academic journals and websites within the specified time frame, and the specific keywords were included in the study.

### The interaction of humans and the environment

Human activities are increasingly affecting the Earth’s climate and ecosystems (IPCC 2007; Zhang et al. 2018). In the late nineteenth century, it was found that a build-up of carbon dioxide in the Earth’s atmosphere could cause an increase in the planet’s surface temperature (Foote 1856). The negative effects of rising temperatures on the environment, biodiversity, and human health are increasingly noticeable and now is scientific evidence that human behaviour is the main cause of climatic changes and global warming (Eyring and Gillett 2023). On the other hand, human behavior is crucial in climate change mitigation, and counteracting the consequences ( 2022).

This human impact has directly or indirectly driven major environmental changes on both a global and local scale (Allamano et al. 2009; Fracasso et al. 2022). Since 1950, to meet the growing needs of the growing human population, the world production of the chemical industry has increased about 50 times. Existing concentrations of chemicals in all components of the environment harm people, wildlife, and ecosystems and chemicals that bioaccumulate can reach toxicological threshold values in sensitive species (Collins et al. 2020). Anthropogenic environmental pollution is indicated as a factor increasing the likelihood of extreme events resulting from climate change (IPCC 2007; Dunlap 2023; Baxter 2002). New synthetic chemicals are created and released into ecosystems. Not all, but some of them are dangerous. Organisms, including humans, cannot avoid the harmful effects and adapt to new environmental conditions. As a result of chemical pollution of the environment, according to WHO, 12.6 million deaths in 2012 were estimated (Prüss-Ustün et al. 2006).

Climate changes are the complex effect of long-term changes in local, global, or regional temperature and weather caused by human activities. The most common manifestations of climate change are an increase in average global temperature, extreme and unpredictable weather, and an increase in the frequency and intensity of natural disasters such as drought, physiological heat stress, fire, storm, river, and coastal flooding as well as the simultaneous occurrence of these factors (Merz et al. 2021; Seneviratne et al. 2023; Shivanna 2022). Shortly, previously unseen combinations of extreme weather phenomena or new, previously unknown ones may occur (Raymond et al. 2020).

Climate change affects every aspect of human life in complex and interconnected ways. The health impacts of climate change can be direct (e.g., exposure to extreme and unusual temperatures, droughts, and floods) or indirect (e.g., through changes in infectious disease epidemiology, vector ecology, and differences in food availability and/or water and air quality). These effects are further magnified by many biological, ecological, and socio-political factors (Neira et al. 2023). In the population as a whole, 23% of all deaths worldwide and 22% of all diseases with the greatest environmental contribution (determined by years of life lost and years lived with disability) are attributable to the environment. There are direct links between environmental conditions and health, such as used energy type, access to safe water and sanitation, healthy, sustainable cities, and climate change (Prüss-Ustün et al. 2006). Some of these factors are related to chemical pollution of the environment, which causes, among others, cardiovascular diseases and lower respiratory tract infections (IARC 2012; Baan et al. 2019; Patel et al. 2020; Naidu et al. 2021). Improving the environment can make a significant contribution to achieving sustainable development goals (SDGs) (Prüss-Ustün et al. 2006). The goals that are directly related to the reduction of environmental pollution of chemical substances are good health and well-being (SDG3), clean water and good sanitation (SDG6), affordable and clean energy (SDG7), sustainable cities and communities (SDG11), responsible consumption and production (SDG12), and climate action (SDG13) (Kęsoń and Gromek 2021a), (Kęsoń and Gromek 2021b).

**Fig. 1.**
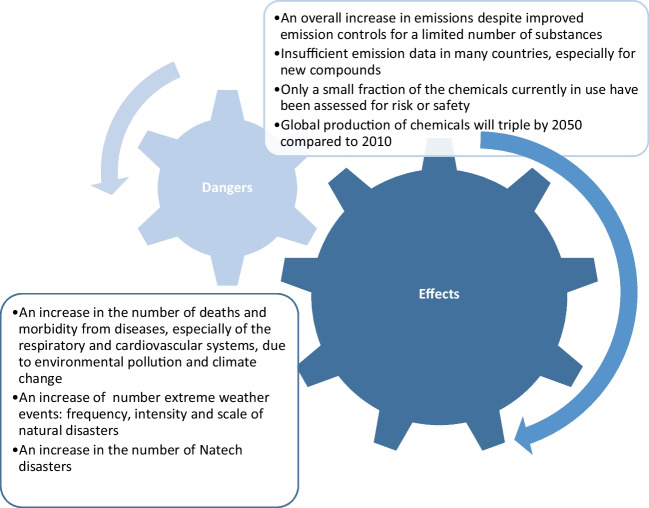
Threats resulting from increasing environmental pollution and their effects

Threats resulting from increasing environmental pollution and their effects are presented in Figure 1.

As estimated by the end of this century, weather-related disasters such as extreme heat, cold spells, wildfires, droughts, floods, and windstorms will affect around two-thirds of the European population annually potentially leading to 50-fold higher fatalities compared to 2017 (Khakzad et al. 2018). Environmental threats such as climate change and air pollution often affect poorer countries disproportionately (Somasundaram et al. 2007). Moreover, future generations will suffer the consequences of pollution they did not cause.

On the other hand, according to the report of the latest Intergovernmental Panel for Climate Change (Seneviratne et al. 2023), human-induced climate change in its current dimension is unprecedented for at least 2000 years and intensifying in every region across the globe (Shivanna 2022). Satellite data show hydroclimatic extreme events are increasing in frequency, duration, and extent under warming conditions (Rohde 2023). The number of catastrophic floods is expected to increase in many regions due to climate and socio-economic changes. Land use transformations such as urbanisation, soil compaction, industrial development, and land pollution can reduce water uptake into the soil and increase soil runoff. Climate change may increase the amount of rainfall and affect snowmelt or catchment humidity, which further increases the risk of flooding and the scale of the disaster (Merz et al. 2021).

An increase in global average temperature can significantly affect the chemical properties, air concentrations, and lifetime of pollutant components. The size and quantity of particulate matter (PM) influence the formation of heat islands and global temperatures. Locally, higher surface temperatures in polluted areas, e.g., heat islands in urban zones or drought-stricken tropical areas, could release regional feedback in atmospheric chemistry and local O_3_ and PM2.5 emissions (Mika et al. 2018).

Due to the complexity of the problem and the interactions of environmental factors, forecasting changes and modelling phenomena are very complicated and burdened with high uncertainty. The number of existing chemical substances, their mixtures, and the number of endpoints in which pollution affects receptors, i.e., people, plants, and animals, mean that their interactions can only be estimated (Watanabe 2017). Additionally, there is a lack of detailed data, e.g., observations of weather conditions and environmental pollution in an appropriate time frame, with the required frequency and density of monitoring points (Mika et al. 2018).

Moreover, a singular planetary boundary for novel entities is not yet quantitatively and qualitatively determined but scientists concluded that the safe threshold has been currently exceeded (Richardson et al. 2023).

Chemical pollution has the potential to cause serious problems in ecosystems and the health of communities at various scales, but it can also change the Earth’s systemic processes on which human life depends. The article reviews the scientific literature on preparing societies (communities) to reduce threats and the consequences of events that we are currently unable to avoid.

### Chemical hazards

Chemical hazards may result from the presence of hazardous substances or their mixtures in the environment due to their use, production, and transport, but they can also be a by-product of such processes. The terminology adopted by the Globally Harmonized System of Classification and Labeling of Chemicals (GHS) (UN 2021) defines substances and mixtures. Substances are chemical elements and their compounds in the natural state or obtained by a manufacturing process, including additives necessary to maintain the stability of the product and impurities deriving from the process used, excluding solvents that can be separated without affecting the stability of the substance or changing its composition. A mixture consists of two or more substances that do not react with each other (UN 2021). Sometimes both chemicals and their mixtures are referred to as chemicals.

Chemicals contribute in many ways to improving our standard of living. However, many chemicals and their mixtures can have properties that make them dangerous and have serious negative impacts on human health and the environment (Bernanke and Köhler 2009; Canipari et al. 2020; Naidu et al. 2021). The classification is based on the type of hazard posed, e.g., explosive, flammable, toxic, corrosive, carcinogenic, or hazardous to the ozone layer (UN 2021).

People are exposed to chemicals at work, in their homes and indirectly through the environment, which can lead to acute or long-term health effects or even death. Hazardous chemicals may be present in products sold to professional users and private consumers for everyday use. The use and subsequent dissemination of hazardous substances can put at the long-term risk use of land and water resources and render, for example, groundwater and fish unfit for human consumption. This, in turn, may have a negative impact on the development of societies. Various measures are therefore necessary to protect human health and the environment from the adverse effects of exposure to hazardous chemicals.

The hazards caused by chemical substances can be considered in different aspects. Contamination can be incidental or a continuous and long-term process. Its effects may be direct or distant in time, or there may be a cumulative effect of exposure to a dangerous substance. The effect of this long-term, often cumulative impact is a negative impact on health, premature deaths, loss of biodiversity, and climate change. Chemical pollutions are the planetary boundary, one of the tipping points of ecosystem collapse, which must not be crossed to protect humanity (Rockström et al. 2009; Persson et al. 2022). A chemical hazard may be caused by the deliberate use of harmful substances, e.g., pesticide use in agriculture or chemical warfare agents. It also can occur as an unintended effect—as a result of a disaster or a permanent effect of some industrial processes, transport, energy, agricultural, and other activities (Naidu et al. 2021), (Suarez-Paba et al. 2019), (Bernanke and Köhler 2009). Moreover, an often overlooked and not included in the statistics cause of releases of chemical substances from installations are system shutdowns related to protection against natural hazards and their subsequent restart. As presented in the publication (Ruckart et al. 2008), more than 72% of the events related to the release of chemical substances were related to natural disasters.

An important gap in the area of methods of removing pollutants from the environment is the approach that takes into account only short- and medium-term effects. For certain hazardous materials, such as substances of very high concern (SVHC), i.e., carcinogenic, mutagenic, or toxic to reproduction (CMR); persistent, bioaccumulative, and toxic (PBT); and very persistent, with strong bioaccumulative properties (vPvB—very persistent and very bioaccumulative) (the European Parliament and the Council 2006), the main problem is long-term, adverse effects for human health and the environment (Necci et al. 2018; Girgin et al. 2019; Krausmann et al. 2019).

### Types of disasters

The catastrophe is the result of a widespread ecological breakdown in the relationship between man and his environment. The term “disaster” refers to an event causing severe losses (SwissRe Institute 2022); it usually occurs suddenly and on such a scale that the affected community requires extraordinary efforts to deal with it locally, and often needs outside or international help (Baxter 2002).

Natural disasters can be the result of earthquakes, volcanic eruptions, landslides, avalanches, floods, tsunamis or droughts, storms and hurricanes, wind action, or fires (Caldera and Wirasinghe 2022). Technical and technological threats always have an anthropogenic origin, and the effects of disasters caused by them can be even more severe than those caused by natural factors. The group of technical disasters includes industrial, nuclear, or transport accidents. When it comes to sociological disasters, we can distinguish two groups: accidental and intentionally caused. In the first case, they appear during mass events that cause a catastrophe for the crowd together with an external event: e.g., the collapse of a building, or a row of seats. In the second case, disasters are related to warfare or terrorist attacks (Provitolo 2009).

Many extreme weather events such as high temperatures causing extensive forest fires, heavy rains and flooding, hurricanes, and storms can lead to technical failures or technological disturbances (Baxter 2002; Santella et al. 2010; Landucci et al. 2014; Khakzad and Van Gelder 2018; Necci et al. 2018; Krausmann et al. 2019; Qin et al. 2020). As a result of such disturbances, hazardous substances are released into the atmosphere or waters. A clear example is the contamination of the Oder River in 2022 (Poland). For many years, there has been the phenomenon of anthropogenic water pollution by many industrial plants, mines, and communication routes located in the Oder basin (Ciazela et al. 2018; Faměra et al. 2021; Kucharski et al. 2022), which has been intensified by the ongoing drought. As a result of the lack of rainfall, the amount of water in the river drastically decreased, which resulted in a higher concentration of pollutants even with the same level of inflowing sewage.

Contamination of the environment and threat to human health or life may also occur during terrorist attacks or warfare as a result of damage to installations, changes in the parameters of technological processes, or damage to infrastructure. All the listed factors triggering the occurrence of a threat are well known and many methods of preventing these events and reacting in the event of their occurrence have been developed and implemented. On the other hand, long-term, slow processes resulting from increasing environmental pollution and leading to climate change are often overlooked or marginalized as disaster risk factors (Booth et al. 2020).

**Fig. 2:**
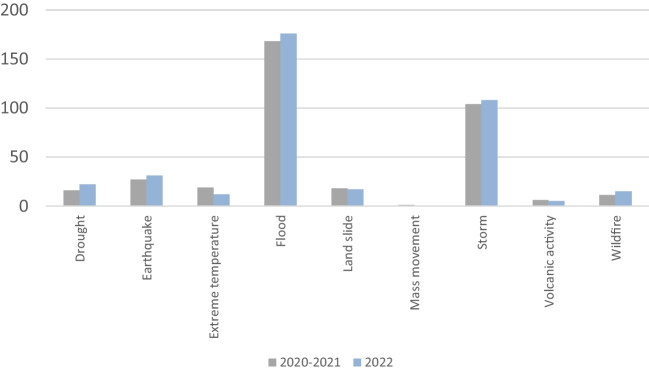
Occurrence by disaster type: 2022 compared to the 2002–2021 annual average (CRED 2023)

In recent years, due to climate change, the frequency and intensity of hurricanes and floods have increased, causing severe damage to urban and industrial areas (Jongman et al. 2012; O’Neill et al. 2017; Qin et al. 2020). In Figure 2, occurrence of natural disasters compared 2022 to 2002–2021 are shown. The phenomena often affect industrialized areas where a large number of oil, chemical, and other industrial facilities are scattered. These plants most often use hazardous substances in production or storage processes, and damage to installations or tanks leads to the release of chemicals. As a result, environmental pollution, fires, and explosions can occur (Krausmann et al. 2019), and even a domino effect (Chen et al. 2019; Dubocq et al. 2020), causing catastrophic consequences to the health or life of the population, damage to the surrounding environment, and huge economic losses (Santella et al. 2010; Griffiths et al. 2018; Qin et al. 2020).

Industrial accidents caused by natural disasters are called natural and technological (Natech) accidents (Girgin et al. 2019). Most research in the field of Natechs has focused on earthquakes due to their destructive nature, although in recent years the focus has shifted to hydro-meteorological hazards (e.g., hurricanes, floods, tsunamis, lightning) (Landucci et al. 2014; Khakzad and Van Gelder 2018; Necci et al. 2018; Suarez-Paba et al. 2019). Despite the risk of forest fires, they are not yet included in the risk assessment of industrial plants. In Europe, the major-accident risk assessment in the preparation of safety reports considers the probability of only a few types of natural hazards: frequent floods and earthquakes (Khakzad et al. 2018). Factors affecting the type and amount of damage caused by weather events mainly result from wind loads, heavy rains, surface and depth of flooding (storms and floods), and lightning (Necci et al. 2018). Some examples of Natech disasters are given in Table 1.

**Table 1:** Selected examples of industrial failures caused by natural disasters

Year	Place	Natural disaster	Technological accident
1971	Czechowice-Dziedzice, Poland	Lightning	At the refinery, lightning struck the breathing chimney of a crude oil tank with a capacity of 12,500 m^3^ of oil. The roof of the tank collapsed and tore the mantle, the fire spread to the roof and walls of the tank, and oil spilt onto the tray. Firefighting water got into the tank, and heated with burning oil, it came to a boil and caused oil to leak over a distance of even over 200 m. In addition, firefighting foam got into the environment (TVP1 2000).
1989	South China Sea.	typhon	Despite that typhoon Gay was quickly approaching, the Seacrest remained in the Gulf of Thailand in drilling mode instead of on the deck-level pipe rack. The force of the wind and waves caused first the vessel to capsize and next the sinking. Due to the vessel overturned, 91 rig workers were killed (ships and oil n.d.).
1994	Milford Haven, UK	lightning	Disruption to the power supply and a loss of process control at a refinery; a release of flammable material caused a fire initiated by a lightning strike and partial shutdown in one area of the refinery (Health and Safety Executive 1997).
1999	Turkey	Earthquake	In refinery Tüpraş, six cylindrical tanks having floating roofs caught fire immediately after the earthquake. Four of them were middle-size tanks and had diameters of 20–25 m and two small-size tanks had a diameter of about 10 m. Naphtha in the middle-size tanks was completely burned. Tanks were damaged as a result of thermal deformation. The fire in an oil or naphtha tank farm was considered to be caused by the bouncing of the floating roof against the inner walls of the tank during the earthquake, causing the generation of sparks that ignited the flammable liquid. The total damage was about 500 million USD (Suzuki 2002).
2000	The Baia Mare and Baia Borsa, Romania	Extreme snowfall combined with exceptionally high temperatures	Overflows of mine tailing dams resulting in peak discharges, and other technical failures caused transboundary heavy cyanide water pollution of the Danube in Romania, Hungary, Serbia, and Bulgaria, finally entering the Black Sea (Greenpeace 2000).
2010	Ajka, Hungary	Rainfall	An industrial accident at a caustic waste reservoir took place at the Ajka aluminium smelter. The northwestern corner of the dam of the tank for waste chemicals, softened and cracked after long-term rain, collapsed, freeing approximately one million cubic meters of liquid waste and flooding nearby localities. Ten people died, and 150 people were injured, initially, about 40 km^2^ of land were affected and the spill reached the Danube River (Day 2010).
2011	Feyzin, France	Lightning	Lightning hit a refinery on a flare and on a tank collecting 2000 m^3^ process water, which contained hydrocarbons. Ignition from lighting in the tank led to an explosion and tank fire. Additionally, the tank did not have a retention basin and firefighting foams reached the Rhône canal via the rainwater network (ARIA Database No. 40953 2011).
2011	Japan	Earthquake and ensuing tsunami	The Fukushima Daiichi nuclear power plant^1^ accident following the tsunami. After the earthquake, to cool the reactor cores, backup diesel generators kicked in which continued to pump water in to cool the reactor cores. But about 1 h later next tsunami wave disabled the backup generators. The next backup system was battery-powered pumps but they could not remove the residual heat still coming from the cores of several reactors. Excess heat caused steam collecting up in the system, which operators eventually vented into the environment along with low concentrations of radioactive elements like caesium and iodin, (Witze 2011).
2017	Texas, USA	Hurricane and the unexpectedly high water levels	The explosions at the chemical plant Arkema were due to the loss of refrigeration to a warehouse storing highly volatile and extremely flammable chemicals (U.S. CSB 2018).
2019	Krasnoyarsk, Russian Federation	Heavy rainfall	A tailings dam breach of a gold mine led to widespread water pollution in the Seiba River (UNECE n.d.).

^1^Nuclear energy facilities are not covered by the Industrial Accidents Convention

Research related to accidents classified as Natech covers a wide range of topics related to risk management and risk assessment, lessons learned from past accidents, event timing, event consequence analysis, and the impact of climate change on Natech risk. Together with the acquired knowledge, steps were taken in the field of Natech risk management, e.g., the California accidental release prevention program (CalARP) or the SEVESO III directive in force in the European Union (Luo et al. 2022), which explicitly requires analysis of external natural hazards to prevent major accidents the inclusion of the Natech risk management guidelines by the Organization for Economic Co-operation and Development (OECD 2021) and the Sendai Framework for Disaster Risk Reduction 2015–2030 call for Natech risk mitigation (UNISDR 2015).

### Risk reduction

Some of the scientific community is paying attention to how global warming is changing the risks associated with certain types of extreme weather conditions (Nascimento and Alencar 2016; Suarez-Paba et al. 2019). This approach is the starting point for developing solutions adapted to changing threats and effective prevention of dangerous events and disaster management (Otto et al. 2018; O’Neill et al. 2017). The classical methodology, i.e., contained in the CCRA guidelines, due to certain specificities, should be adapted to the risks associated with climate change (Wolanin et al. 2022).

Chemical emissions can result in serious incidents and even disasters. Factors such as chemical properties, emission volume, meteorological conditions, the roughness of surface involvement, and the emitter environment affect the scale and type of actions taken aimed at reducing the effects of hazards (Wood and Fabbri 2019). Knowledge of the properties of substances is the key to their safe acquisition, production, use, and waste management. Many of the risk management methods in this area are preventive in nature, as they contribute to preventing exposure to chemicals during manufacturing or industrial use. Plants producing, processing, and storing chemicals are critical infrastructure facilities. The term “critical infrastructure” is connected to resources that are essential for society to maintain health, safety and security, as well as the economic and social well-being of people (UE 2008).

Solving the problem of chemical hazards causing Natech disasters is usually a big challenge that requires collecting the necessary information, analyzing it, drawing conclusions, and taking action in the field of developing legal, organizational, or technological regulations to direct preventive actions and prepare response plans These areas should interpenetrate and complement each other.

**Fig. 3.**
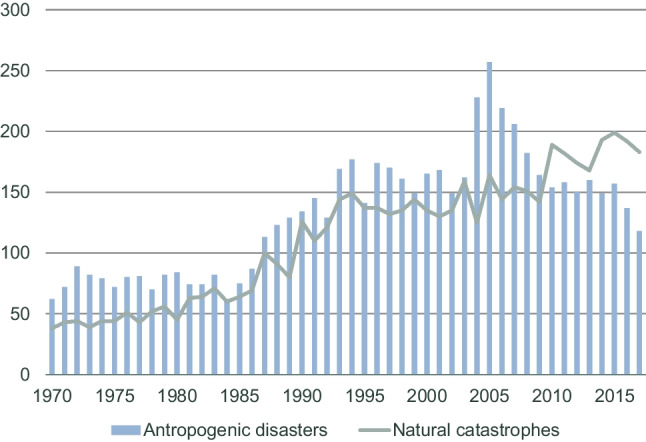
Number of catastrophic events, 1970–2017. *Source*: Swiss Re Institute

Figure 3 shows the number of catastrophic events in the world from 1970 to 2017. Thanks to the development of technology, organizational changes, and legal regulations, the number of technical failures and accidents in the world shows a downward trend, while the number of natural disasters is constantly growing (Swiss Re Institute 2018). Progressive climate change generates more and more serious and long-lasting infrastructural damage (Salimi and Al-Ghamdi 2020); therefore, resilience and protection of critical infrastructure currently are becoming increasingly important. Increasingly, the damage is the result of natural disasters or multiple disasters. However, the growing complexity of critical infrastructure, despite the development of systems for disaster information and supporting decisions that enhance the ability to cope with natural disasters, suggests that this type of situation will intensify (Gromek 2021). Despite advances in knowledge, the emergence of threats exceeds the possibilities of limiting them. The best defence against future disasters is addressing climate change, and reducing the vulnerability and exposure that drive disasters (United Nations Office for Disaster Risk Reduction 2022). On the one hand, we should strive to reduce pollution; on the other hand, we should improve the system of preventing disasters and limiting their effects (UNISDR 2015).

### Disaster databases

Conducting observations, their analysis and conclusions from experience are one of the methods of learning and development. Societies can benefit from large sets of data collected over a long period, by different groups and in different areas. Comprehensive historical data stored in disaster databases is critical to a variety of risk reduction tools, such as setting up disaster risk assessment models, preparing disaster management plans, and conducting disaster risk mitigation cost analysis. Several global or local disaster databases have been created so far: Emergency Events Database (EM-DAT), Failure and Accidents Technical information System (FACTS), eNatech, NatCatSERVICE, Major Accidents Reporting Systems (MARS), Sigma, Global Facility for Disaster Reduction and Recovery (GFDRR), and CATNAT (Jones et al. 2022; Luo et al. 2022; Official Journal of the European Communities 1982; Mazhin et al. 2021). Undoubtedly, the existence of such systems is valuable, but inconsistencies in data collection and methodological difficulties in quantifying their effects are their weaknesses. As a result, the range and reliability of statistical conclusions that can be drawn from disaster data are limited (Jones et al. 2022; Wood and Fabbri 2019). The strengths and limitations of such systems result from, among others (Wood and Fabbri 2019; Mazhin et al. 2021), the following:

- fatalities are always recorded
- low precision in determining the number of victims and the severity of injuries
- lack of data on injuries or acute exposure with long-term effects on mental health, long-term effects on the local economy and social life
- use various measures to describe the impact on the environment, e.g., flooded area, river length, and duration of a power outage
- lack of data on cleanup and restoration costs or resource loss costs
- data on property damages are usually limited to the site; in the case of large incidents, data can also be found in annual insurance reports
- registered the economic impact of temporary and permanent closures of production lines and plants
- contain data on social disruption, but the effects as duration, location, and the size of the population they affect are poorly described
- no database takes climate change into account

In addition, while these databases were designed to store data on past chemical accidents to support inference, none of them can be used to directly analyze the spatial distribution of historical Natech events. Moreover, for chemical hazards, a system to track the frequency and severity of incidents over time is insufficient to assess the risk of an accident. The occurrence of a chemical accident risk may be underestimated, as major-accident-free periods do not necessarily result from risk mitigation but from a statistical distribution. Serious chemical accidents are relatively rare and have a very diverse background in terms of the nature, circumstances, and dynamics of the occurring damage. In addition, data aggregation masks large differences in the causes of chemical accidents and exposure to accident risk across industries and geographies (Jones et al. 2022; Wood and Fabbri 2019)**.**

### Lessons learned

Lessons learned from the analysis of a series of industrial disasters in the 1970s and early 1980s led to international initiatives and new regulations to prevent large-scale releases of chemicals and their mixtures (Official Journal of the European Communities 1982). Higher awareness and control mechanisms are effective in preventing similar extreme events from reoccurring elsewhere. This applies both to sudden or immediate states of danger resulting from the extraction of raw materials, transport to places of processing and use, processing, use and disposal of substances, mixtures or products, as well as long-term and increasing phenomena leading to a climate disaster.

The analysis of event data and the formulation of conclusions made it possible to develop legal, organizational, and technical or technological solutions that increased the safety of industrial facilities and gave grounds for preparing services and communities to limit and eliminate the effects of releasing chemical substances or their mixtures. Global trends in urbanization and climate change have increased the need for more developed disaster response systems (Garschagen and Romero-Lankao 2015; Zhang et al. 2018). In practice, however, emergency planning has rarely taken climate change into account (Arnell 2022). Solutions adapted to local or regional conditions operate in, e.g., China (Spencer et al. 2019), India, the USA, and European Union (Margus et al. 2023). But climate change is an ongoing process, so preparation for its consequences should be ongoing. The European Union only in 2022 called for adapting civil protection systems to the effects of climate change, without specifying in detail how these systems should be adapted (Council of the European Union 2022).

Emergency planning is an element of disaster risk reduction that deals in particular with the preparation for events and the immediate removal of their effects. For certain types of environmental hazards—flooding, drought, and erosion, for example—it is often possible to reduce the exposure and vulnerability of people and property through technical solutions such as protective barriers, spatial planning, and building codes. For others—heat, cold, and storms—exposure and vulnerability can only be truly reduced through codes, standards, and measures to support recovery or operation.

To prevent and reduce the effects of an industrial accident, internal and external operational and rescue plans are developed. According to Seveso III Directive to prevent major accidents, limit the consequences, and prepare emergency plans and response measures, the operator should, in the case of establishments where dangerous substances are present in significant quantities, submit information to the competent authority in the form of a safety report. It should be emphasized that the Directive requires that the increased risk of a major accident by the probability of natural disasters related to the location of the plant be taken into account when preparing major accident scenarios. The SEVESO III Directive also imposes an obligation to inform those concerned what to do in the event of a pollution accident (Directive 2012/18/EU 2012). The importance of contingency planning for natural disaster risk reduction, therefore, varies according to the type of hazard. In the case of Natech disasters, adapting rescue plans as a result of estimating the threats caused by natural factors resulting from climate change, is based on analysis of data collected in databases (Jones et al. 2022), especially in terms of information on existing threats.

While risk assessment is largely based on interdisciplinary knowledge, further elements of risk management are often driven by economic, technical, social, or political considerations (Monte et al. 2021).

### Policies

Refraining from systematic or unreasonable harm is fulfilled by commitments, policies, and practices that aim to anticipate pollution effects associated with the use of chemicals and to compensate for unforeseen damage caused by pollution. These commitments are connected to reducing emissions of chemicals into the environment and removing from use chemicals that bioaccumulate. An example of such an approach is the Directive on National Emission Reduction Commitments (NEC) (Zhang et al. 2021; European Parliament and Council 2016), which from 2020 applies to emission ceilings for five types of air pollutants that have a significant negative impact on human health and the environment. These substances are nitrogen oxides (NO_x_), non-methane volatile organic compounds (NMVOC), ammonia (NH_3_), sulfur dioxide (SO_2_), and particulate matter with a particle size of not more than 2.5 μm (PM2.5). The NEC provisions aim to reduce the number of premature deaths caused by air pollution by 55% compared to 2005 levels, and the threat to the biodiversity of EU ecosystems by 25%.

The sources of emissions can be chemical production plants, but emissions also occur at further stages of the life cycle of chemical substances and mixtures: transport, processing, use, and disposal of waste. Therefore, limiting the impact of chemicals on people and the environment may require actions related to decisions regarding their placing on the market (The European Parliament and the Council 2006) or, for example, reducing emissions from a specific plant. For the vast majority of chemicals, effective solutions result from the obligations imposed on actors in the supply chain to communicate and use the information provided following the Globally Harmonized System of Classification and Labeling of Chemicals (GHS) (UN 2021). Efforts by governments and industries to develop methods to manage the risks of chemicals are supported by international programs (OECD 2021). Risk management strategies for mitigating and adapting to climate change propose to reduce GHG emissions and cool the city through changes in the built environment, land use, and transportation. Risk management strategies for climate change mitigation and adaptation propose reducing greenhouse gas emissions and cooling the city through changes in land use: built environment, land use, and transport (Harlan and Ruddell 2011).

Using more green chemistry to produce ecological chemicals and monitoring the reciprocal interactions between chemicals and wildlife are not only a manifestation of responsibility for the environment but also an effective way to avoid penalties for environmental damage. The implementation of recycling technologies and the reuse of chemicals prevent further accumulation of chemicals and waste in the environment and due to the saving of raw materials is economically justified.

Advances in technology can reduce risks to the ecosystem and human health by reducing bioaccumulation and toxicity. Law regulations and policies should allow the sale only of products and chemicals that can be recycled and encourage investment in technological advances in processing waste (Collins et al. 2020).

### Technological progress

Traditionally, contamination control is often done at the end of the pipe, but preventing contamination upstream in the supply chain offers the potential for additional environmental and health benefits. The sooner action is taken to reduce the negative effects of chemicals, the lower the cost to society as well as to industry, as it is much less complicated to take early action. Choosing a preventive approach not only saves human and natural resources but also often brings financial benefits.

One method of risk mitigation is the ability to select alternative substances or technologies that present a lower risk for a particular chemical or a particular use of that chemical. Another solution is to change the production technology of the product or to use other methods to eliminate releases to the air and water from production plants. A set of such solutions was postulated about 30 years ago as a set of principles called “green chemistry” (Anastas and Eghbali 2010).

As with any risk reduction procedure, for chemical hazards, the following should be considered when selecting the most appropriate tool:

- degree of threat and risk
- efficiency
- durability over time
- costs and benefits for various entities
- socio-economic consequences
- administrative burden

To achieve the expected result, i.e., eliminating or reducing the risk to human health and the environment posed by a chemical substance, the different benefits and costs of using these tools should be weighed up and their synergistic effects should be taken into account. One possibility is to introduce an administrative (legal) measure, such as a total ban or restriction of certain uses (UN Treaty Series 1989), (UNEP 2019). Another solution is to investigate whether economic arguments such as taxes or fees, alone or in combination with other mechanisms, will be used to reduce the risk. Yet another option is to assess whether an information instrument such as government-industry dialogues can be sufficient to mitigate the risk to the extent required.

When the measures taken by industry are not sufficient to reduce the risk and there is a need to further reduce environmental and human exposure to a chemical, additional risk mitigation measures are required. Then, the preferred method of reducing the risk may be a complete ban on the use or its limitation to critical areas, e.g., substances that are very persistent and very bioaccumulative (vPvB) or substances that are carcinogenic, mutagenic, or toxic to reproduction (CMR) (UN Treaty Series 1989), (UNEP 2019).

## Conclusions

The impact of chemical pollution on human health and the condition of the natural environment is not only a very important issue but also an extremely complex one. Chemical pollution is the result of human activities that are increasingly affecting the Earth’s climate and ecosystems.

The amount and type of pollutants released into the air, water, and soil exceed the possibilities of adaptation and self-cleaning of the environment.

The main problem in reducing the amount of pollution in the environment is striking a balance between economic growth and environmental protection. The possibility of reconciling human activities with the preservation of the stability of the Earth’s biophysical systems raises serious concerns. What’s more, chemical pollution of the environment has been included in the planetary boundaries, the crossing of which threatens the collapse of the Earth’s ecosystem.

Anthropogenic environmental pollution leads to climate change and is increasingly mentioned as a factor that multiplied the likelihood of extreme events. Unpreparedness for natural disasters transforms hazards such as floods, wildland fires, lighting, and tsunamis into catastrophic technical events causing human, environmental, and economic losses.

Growing technological possibilities allow for increasing the safety of technological processes, reducing raw material consumption and pollutant emissions. Spatial development planning is of great importance for protection against emerging emergency releases of pollution and mitigation of local threats. It is a priority to take into account the impact of extreme natural phenomena on Natech’s threats and to prepare adequate plans and procedures in the field of civil protection.

Prevention of chemical threats is also implemented by making commitments to reduce emissions of substances with a large impact on human health and life expectancy and ecosystems. These provisions are intended to reduce the number of premature deaths and diseases caused by air pollution, as well as to reduce the threat to the biodiversity of ecosystems.

Increased preparedness for disasters posed due to climate changes is critical for society’s safety and requires contingency plans to continually adapt to the changing frequency, intensity, and scale of natural disasters.
